# Preparation of SiO_2_-Protecting Metallic Fe Nanoparticle/SiO_2_ Composite Spheres for Biomedical Application

**DOI:** 10.3390/ma8115416

**Published:** 2015-11-13

**Authors:** Pin-Wei Hsieh, Ching-Li Tseng, Dong-Hau Kuo

**Affiliations:** 1 Department of Materials Science and Engineering, National Taiwan University of Science and Technology, No. 43, Sec. 4, Keelung Road, Taipei 10607, Taiwan; wisepowerflow@gmail.com (P.-W.H.); dhkuo@mail.ntust.edu.tw (D.-H.K.); 2 Graduate Institute of Biomedical Materials and Tissue Engineering, College of Biomedical Engineering, Taipei Medical University, No. 250, Wu-Hsing Street, Taipei 110, Taiwan

**Keywords:** metallic iron, SiO_2_, nanoparticle, magnetic property, drug carriers

## Abstract

Functionalized Fe nanoparticles (NPs) have played an important role in biomedical applications. In this study, metallic Fe NPs were deposited on SiO_2_ spheres to form a Fe/SiO_2_ composite. To protect the Fe from oxidation, a thin SiO_2_ layer was coated on the Fe/SiO_2_ spheres thereafter. The size and morphology of the SiO_2_@Fe/SiO_2_ composite spheres were examined by transmission electron microscopy (TEM). The iron form and its content and magnetic properties were examined by X-ray diffraction (XRD), inductively-coupled plasma mass spectrometry (ICP-MS) and a superconducting quantum interference device (SQUID). The biocompatibility of the SiO_2_@Fe/SiO_2_ composite spheres was examined by Cell Counting Kit-8 (CCK-8) and lactate dehydrogenase (LDH) tests. The intracellular distribution of the SiO_2_@Fe/SiO_2_ composite spheres was observed using TEM. XRD analysis revealed the formation of metallic iron on the surface of the SiO_2_ spheres. According to the ICP-MS and SQUID results, using 0.375 M FeCl_3_·6H_2_O for Fe NPs synthesis resulted in the highest iron content and magnetization of the SiO_2_@Fe/SiO_2_ spheres. Using a dye loading experiment, a slow release of a fluorescence dye from SiO_2_@Fe/SiO_2_ composite spheres was confirmed. The SiO_2_@Fe/SiO_2_ composite spheres co-cultured with L929 cells exhibit biocompatibility at concentrations <16.25 µg/mL. The TEM images show that the SiO_2_@Fe/SiO_2_ composite spheres were uptaken into the cytoplasm and retained in the endosome. The above results demonstrate that the SiO_2_@Fe/SiO_2_ composite spheres could be used as a multi-functional agent, such as a magnetic resonance imaging (MRI) contrast agent or drug carriers in biomedical applications.

## 1. Introduction

Magnetic nanoparticles (MNPs) exhibit superparamagnetic behavior, as they contain a single dipole in a single domain due to the size effect, which can be aligned under an applied external magnetic field [1]. MNPs show potential biomedical applications, such as localized cellular therapy, magnetically-guided drug delivery, magnetic resonance imaging (MRI), hyperthermia treatment, and magnetofection on the interaction with an external magnetic field [1,2]. Among MNPs, metallic iron nanoparticles (Fe NPs) have been studied and applied in the research fields of optics, catalysis, magnetism and biomedicine for years [3,4,5]. One benefit to prepare metallic iron NPs is that FeNPs have a much stronger shortening effect on T_2_ relaxation time than iron oxide nanoparticles (IONPs), suggesting that FeNPs may be more effective MRI contrast agents [3]. The synthesis of Fe NPs with a uniform particle size distribution has been demonstrated using high-temperature thermolysis in oil-based reaction systems [6,7]. However, these reactions were considered harmful because of the involvement of toxic reagents and the high energy consumption. From the viewpoint of practical applications, any method to fabricate Fe NPs must take into account the susceptibility of Fe NPs to oxidation and aggregation owing to the magnetic interaction between the particles. Controlling the oxidation of Fe NPs is particularly important, as oxidation leads to a degradation in their magnetic properties [3,8]. Since Fe NPs are oxidized rapidly in water and air, they exhibit a loss or decrease in magnetism and dispersibility in a biological environment. Therefore, preserving the stability of Fe NPs is considered another challenge for biomedical applications.

To prevent the oxidation of Fe NPs, the core-shell structures that are stable, biocompatible and hydrophilic were demonstrated using surface modifications with organic polymers of poly(acrylic acid) [9], poly(ethylene glycol) [10], dendrimers [11], chitosan [12,13] and silica (SiO_2_) layers [7,8]. The SiO_2_ shells provide an electrically-insulting layer that decreases energy loss and additionally prevents the possibility of a decrease in permeability due to Fe oxidation [7]. Li *et al.* prepared α-Fe nanoparticle/ordered mesoporous silica with the aid of a triblock copolymer using oxidation and reduction reactions [14]. Yang *et al.* formed Fe-core/SiO_2_ shell nanoparticles using oleic acid and citric acid as the surface capping agents in an aqueous environment at room temperature [7]. However, in this case, a 400 °C reduction treatment was needed to obtain pure Fe/SiO_2_ nanoparticles without FeO*_x_*, increasing the energy uptake.

**Figure 1:**
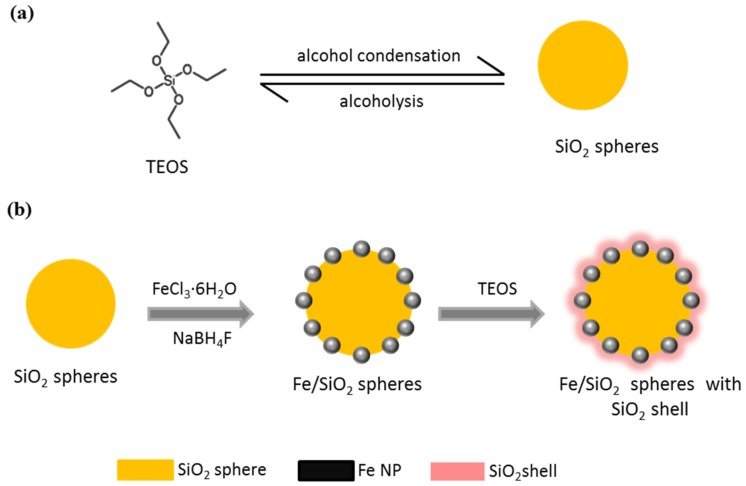
Diagram outlining the synthesis process of Fe/SiO_2_ spheres with the SiO_2_ shell (abbreviated as SiO_2_@Fe/SiO_2_ composite spheres): (**a**) the SiO_2_ spheres were prepared by tetraethyl orthosilicate (TEOS) condensation; (**b**) the formation of metallic Fe-containing nanoparticles (Fe NPs) on the SiO_2_ spheres by the addition of iron chloride hexahydrate (FeCl_3_·6H_2_O) in the SiO_2_-sphere suspension as the iron source, reduced with sodium borohydride (NaBH_4_) and followed by TEOS addition again for coating SiO_2_ shells to prevent the oxidation of metallic Fe NPs on the shells.

The aim of this study was to prepare metallic Fe NPs on SiO_2_ spheres, further protected by a SiO_2_ shell (SiO_2_@Fe/SiO_2_) to prevent iron oxidation using a sol-gel process at low heat treatment conditions, as per the schematic shown in Figure 1. As a result, multifunctional and biocompatible magnetic particles were produced that could be used as an MRI contrast agent, in hyperthermia treatment or as a magnetically-guided drug carrier in biomedical applications.

## 2. Experimental Section

### 2.1. Preparation of SiO_2_@Fe/SiO_2_ Composite Spheres

SiO_2_ spheres were prepared using the modified Stöber method with the addition of dodecane. The detailed procedure is provided in our previous publication [15]. Briefly, a mixture of 10 mL of anhydrous ethanol, 0.75 mL of tetraethyl orthosilicate (TEOS, 99.999%, Sigma-Aldrich, St. Louis, MO, USA), 6 g of dodecane and 2 mL of water was stirred at room temperature for 30 min followed by the addition of 0.4 mL of ammonia solution and further stirring for 10 min. Then, 6 mL of anhydrous ethanol were added to this mixture, and the stirring was continued for 2 h at room temperature. Then, the obtained SiO_2_ spheres were separated by centrifugation (1500 rpm, 20 min) and washed three times with ethanol. Finally, the SiO_2_ spheres were dried under vacuum. The dried SiO_2_ spheres were further fired at 300 °C for 3 h to eliminate any residual dodecane. Next, Fe^3+^ ions were deposited onto the SiO_2_ spheres. To do this, iron chloride hexahydrate (FeCl_3_·6H_2_O, Sigma-Aldrich) solution of three different concentrations (0.1 M, 0.375 M and 0.51 M) was prepared by dissolving the appropriate amount (0.234g, 0.912 g and 1.285 g, respectively) in a mixture of SiO_2_ sphere (1.5 g) solution of 7.2 mL of anhydrous ethanol and 1.8 mL of de-ionized water. After Fe^3+^ adsorption and ultra-sonication at regular intervals for 3 h, the Fe^3+^-adsorbed SiO_2_ spheres were separated from the solution by centrifugation (1500 rpm, 20 mines). To prevent the escape of Fe^3+^ during the subsequent reduction reaction, the Fe^3+^-adsorbed SiO_2_ spheres were dispersed in a 5-mL dodecane oil-phase solvent. Then, Fe-NPs-deposited SiO_2_ spheres were prepared by reducing the Fe^3+^ ions adsorbed on the SiO_2_ spheres. Sodium borohydride (NaBH_4_) aqueous solutions (5 mL) with concentrations of 0.10 M, 0.15 M and 0.18 M were used as the reducing agent to form Fe NPs on the SiO_2_ spheres fabricated using 0.1 M, 0.375 M and 0.51 M FeCl_3_·6H_2_O solutions, respectively. To prevent the oxidation of the Fe NPs, the Fe/SiO_2_ spheres were covered with a thin SiO_2_ layer, which was obtained by immersing 0.1 g of Fe/SiO_2_ spheres in a solution comprising 30 mL of anhydrous ethanol and 0.06 g of TEOS followed by the addition of 0.086 mL of 25%–30% ammonium hydroxide and stirring for 2 h. After rinsing and drying, the prepared powders were stored under anhydrous ethanol. The three kinds of prepared powders were denoted as SiO_2_@Fe/SiO_2_-0.1, SiO_2_@Fe/SiO_2_-0.375 and SiO_2_@Fe/SiO_2_-0.51 depending on the amount of FeCl_3_·6H_2_O used.

### 2.2. Characterization and Measurements of SiO_2_@Fe/SiO_2_ Composite Spheres

The morphology of the magnetic SiO_2_@Fe/SiO_2_ composite spheres was observed using a transmission electron microscope (TEM) (H-7000, equipped with a CCD camera, Hitachi, Tokyo, Japan). Mass spectroscopy with an inductively-coupled plasma mass spectrometer (ICP-MS, X Series II, Thermo Scientific, Waltham, MA, USA) was used to quantitatively analyze the Fe content on the Fe/SiO_2_ composite spheres after they were digested in nitric acid solution. Powder X-ray diffraction (PXRD) data were obtained on a D2-phaser diffractometer (D2PHASER) (Bruker, Billerica, MA, USA) using CuKα radiation (λ = 1.5418 Å). The magnetic properties of the particles were analyzed using a quantum design magnetic property measurement system Model SVSM 067 (Quantum Design, San Diego, CA, USA) at 300 K (room temperature). The same mass (10 mg) of matter was used for all of the samples.

### 2.3. Dye Released from SiO_2_@Fe/SiO_2_ Composite Spheres

The dried SiO_2_@Fe/SiO_2_ composite spheres loaded with fluorescent dye (fluorescein isothiocyanate (FITC), Sigma-Aldrich) were suspended in phosphate buffer saline (PBS, pH 7.4, 1 mL) to simulate drug release behavior from the carriers. These dye-containing spheres were soaked in PBS (40 mL) and shaken at a constant rate of 140 rpm at 37 °C. At given time points, 1 mL of the released buffer was extracted for analysis, and 1 mL of fresh PBS was added into the system to maintain the same volume of release medium. The dye concentration was analyzed using a multifunctional microplate reader (Varioskan Flash, Thermo Scientific, Waltham, MA, USA).

### 2.4. In Vitro Evaluation of SiO_2_@Fe/SiO_2_ Nanoparticles

Cell viability assay: The mouse fibroblast cell line (L929) was cultured in Modified Eagle’s Medium (MEM) supplemented with 10% fetal bovine serum (FBS), 100 U/mL penicillin and 100 μg/mL streptomycin at 37 °C under a 5% CO_2_ atmosphere. Reagents for cell culture were obtained from Gibco BRL (Gaithersburg, MD, USA). Each well of the 96-well culture plate was seeded with 5 × 10^3^ L929 cells. The cells were cultivated overnight in 200 μL of culture medium, which then were mixed with SiO_2_@Fe/SiO_2_ composite spheres at different concentrations (from 15.6 to 1000 µg/mL). Cells incubated in the culture medium without SiO_2_@Fe/SiO_2_ composite spheres were used as the control group. After 1 and 3 days of cultivation, the cultured cells were analyzed using the cell proliferation reagent kit (Cell Counting Kit-8 (CCK-8), Sigma-Aldrich). The cell proliferation assay was carried out by adding the WST-8 reagent ([2-(2-methoxy-4-nitrophenyl)-3-(4-nitrophenyl)-5-(2, 4-disulfophenyl)-2H-tetrazolium, mono- sodium salt]). After incubation with WST-8 for 3 h at 37 °C, the amount of formazan dye, generated by the activity of the dehydrogenases contained in the cells, was proportional to the number of living cells; the absorbance at 450 nm was measured using a microplate reader (Multiskan GO, Thermo). All experiments were repeated 6 times for statistical analysis. The cells were stained with a Live/Dead Cell Double staining kit (Sigma-Aldrich) to observe cell viability; the live cells emit green fluorescence, and the dead cells emit red fluorescence. Images were acquired using an inverted fluorescence microscope (Nikon, TiS, Tokyo, Japan) and were analyzed using Nikon NIS Element software.

Cytotoxicity assay: A lactate dehydrogenase (LDH) assay was used to evaluate cell damage (*i.e.*, cytotoxicity) induced by the SiO_2_@Fe/SiO_2_ spheres. LDH is a stable cytosolic enzyme from mitochondria that is released from the cell when it is lysed. The supernatant acquired from the cell culture medium in the cell viability test was collected for the LDH assay. LDH was measured using a commercial assay kit (CytoTox 96 Non-Radioactive Cytotoxicity Assay, Promega, Madison, WI, USA).

Intracellular distribution of particles: The cells were cultured with SiO_2_@Fe/SiO_2_ nanospheres for 2 h and then washed with 7% sucrose in a 0.1 M sodium cacodylate buffer (pH 7.4) to remove excess particles. After that, the cells were fixed in 2% paraformaldehyde and 2.5% glutaraldehyde at 4 °C for 1 h and then postfixed in 10% osmium in cacodylate buffer. Finally, they were dehydrated and embedded in spur resin. Blocks were sectioned using a Leica ultracut UCT ultramicrotome with a diamond knife. The sections were examined under a TEM (HT-7700, Hitachi, Tokyo, Japan).

## 3. Results and Discussion

### 3.1. Characterization of SiO_2_@Fe/SiO_2_ Composite Spheres

#### 3.1.1. Elemental Composition

The SiO_2_@Fe/SiO_2_ composite spheres were synthesized as a carrier for magnetic drug targeting by using the sol-gel route and a chemical reduction method. The ICP-MS composition analysis data for the Fe content in the SiO_2_@Fe/SiO_2_ composite spheres are summarized in Table 1. The content of Fe in the SiO_2_@Fe/SiO_2_ composite spheres was determined to be 0.59 wt%, 2.42 wt% and 2.49 wt%. With increasing FeCl_3_ concentration, the amount of Fe deposited increased, but reached saturation at (FeCl_3_) ≥ 0.375 M.

**Table 1:** ICP-MS analysis results for the Fe content in the SiO_2_@Fe/SiO_2_ composite spheres prepared with different concentrations of iron(III) chloride hexahydrate.

FeCl_3_·6H_2_O Concentration	Fe (mg) by ICP-MS	Fe wt% by ICP-MS
0.100 M	0.0214	0.59
0.375 M	0.0828	2.42
0.510 M	0.0785	2.49

#### 3.1.2. Iron Nanoparticle Type and Morphology

**Figure 2:**
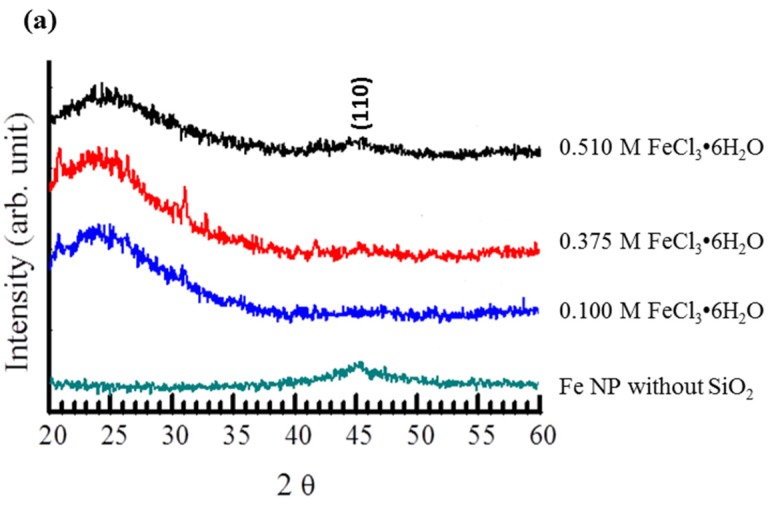

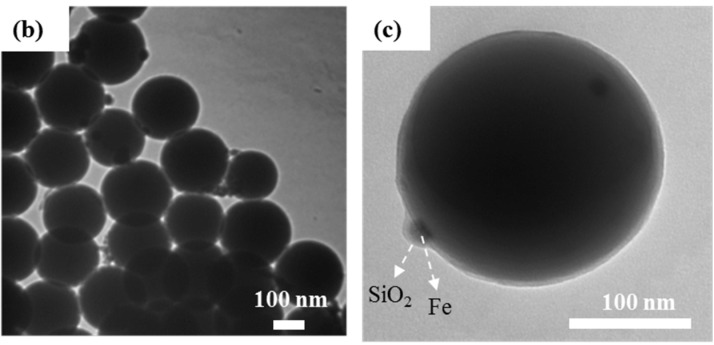
(**a**) XRD diffraction patterns of the SiO_2_@Fe/SiO_2_ composite spheres prepared at different concentrations of FeCl_3_·6H_2_O. Fe NPs prepared under the SiO_2_ sphere-free condition were also examined for comparison purposes. (**b**) TEM images of the SiO_2_@Fe/SiO_2_ composite spheres prepared at an iron chloride hexahydrate concentration of 0.375 M; and (**c**) the higher-magnification image of these spheres.

Figure 2a shows the XRD diffraction patterns of the SiO_2_@Fe/SiO_2_ composite spheres for different concentrations of the Fe precursor. According to the standard iron (Fe) metal XRD pattern (JCPDS data No. 06-0696), the 2θ angle corresponding to the (110) lattice planes of bcc iron should appear at 45.1° [4,5,16]. The pure Fe NPs prepared in this experiment exhibit a weak diffraction peak at about 45°, and this can be attributed to the contribution of Fe (110) with a small size. Mustapic *et al.* reported an overlap of Fe (110) and Fe_2_B (211) peaks at 45° [17]. However, they also reported that the Fe_2_B peak was sharper than the Fe (110) peak [17]. The fact that the peak at 45° is broad could be attributed to the small size of the Fe particles, so we think that this peak is mainly due to Fe, although iron borides could possibly exist in very low amounts. In this work, the Fe nanoparticles (Fe NPs) were deposited on bigger SiO_2_ spheres. SiO_2_ is the major phase, and Fe NPs correspond to a minor phase. Therefore, it is difficult to observe the crystallization of the Fe NPs on SiO_2_. Martinez *et al.* prepared amorphous SiO_2_ by a sol-gel procedure and observed in the XRD pattern a broad peak around 2θ = 21.8° corresponding to amorphous SiO_2_ [8]. Therefore, the peak at 2θ = 21° may result from the amorphous SiO_2_ layer. The peaks at 21.5°, 31.8° and 32.7° might be mistakenly thought of as Fe_3_O_4_. However, Fe_3_O_4_ needs Fe^2+^ and Fe^3+^ species to coexist and high temperature pyrolyzation to form. With only the FeCl_3_·6H_2_O and lower heating temperature, it is not possible to form Fe_3_O_4_. The peaks at 31.8° and 32.7° could possibly correspond to the (331) and (420) peaks of NaCl, which was formed as a byproduct after reaction between NaBH_4_ and FeCl_3_·6H_2_O. For the SiO_2_@Fe/SiO_2_ composite spheres, the broad maximum around 25°–28° probably arises owing to the presence of amorphous silica [18] and the incomplete crystallization of SiO_2_@Fe/SiO_2_, which was a result of the low heating temperature used (300 °C). TEM images of the SiO_2_@Fe/SiO_2_ composite spheres treated with 0.375 M FeCl_3_·6H_2_O solution are shown in Figure 2b. The Fe^3+^ ion in the solution was expected to penetrate into the SiO_2_ shell and remained trapped after being reduced. No aggregations were observed in the group of SiO_2_@Fe/SiO_2_-0.0.1 composite spheres, which were synthesized at a lower Fe^3+^ ion solution. With increasing Fe^3+^ content, Fe aggregates were observed on the surfaces of the SiO_2_@Fe/SiO_2_-0.375 and SiO_2_@Fe/SiO_2_-0.51 spheres. Figure 2c shows the TEM image of SiO_2_@Fe/SiO_2_-0.375 spheres after the surface was covered by a sol-gel SiO_2_ thin layer; the 10 nm Fe NPs formed by aggregation are observable in the TEM image. The non-aggregated Fe NPs that were trapped in the pores of the SiO_2_ particles, on the other hand, cannot be observed.

#### 3.1.3. Magnetic Properties

**Figure 3:**
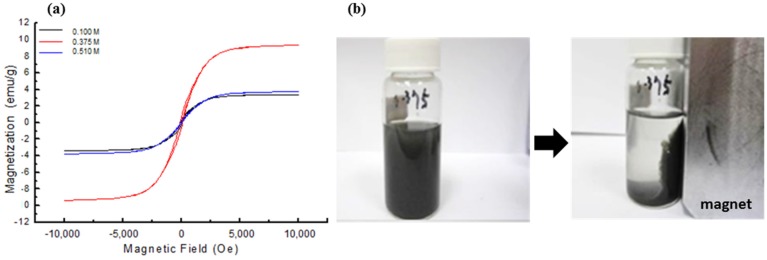
(**a**) The M-H hysteresis loops of SiO_2_@Fe/SiO_2_ spheres prepared with Fe precursor (FeCl_3_·6H_2_O) at 0.1 M, 0.375 M and 0.51 M; (**b**) photographs of the SiO_2_@Fe/SiO_2_ composite (0.375 M) in an aqueous suspension, which appears black in color. Almost all of the particles are attracted to the magnet (right panel), with the aqueous solution now appearing clear, as the particles are up against the side of the vial.

Figure 3a shows the magnetization (M)—magnetic field (H) hysteresis loops of SiO_2_@Fe/SiO_2_ composite spheres prepared at different concentrations of the Fe precursor. To confirm the anti-oxidation effect of the SiO_2_ thin layer, this test was performed after seven days of composite sphere preparation. The saturation magnetization (Ms) values are 3.34, 9.37 and 3.74 emu/g for the individual composite particles synthesized using different concentrations of FeCl_3_·6H_2_O. Fe NPs can be easily oxidized to non-magnetic iron oxide in the presence of oxygen and water. Kim *et al.* used mesoporous silica as an antioxidative layer for protecting metallic iron. However, the Ms value decreased by about 37% from 1.67 emu/g to 1.06 emu/g after seven days, due to the oxidation of Fe NPs [19]. The data contradict this statement that the product produced in 0.375 M FeCl_3_·6H_2_O has superior magnetic properties as compared to that synthesized at 0.1 M and 0.51 M. FeCl_3_·6H_2_O at 0.375 M might be the optimal condition to reach the maximum of the attached Fe content. Standard crystalline bcc Fe bulk has an Ms value of 222 emu/g (Fe) at 298 K [20] and a critical grain size of 15 nm for superparamagnetism [21]. It was reported that superparamagnetic iron oxide nanoparticles (SPION) display relatively high Ms at a high field (67 emu/g at 10 kOe), but after coating with a silica layer (silica–SPION), its Ms decreases to 30 emu/g mainly due to dilution with the nonmagnetic phase [22]. Our Fe NPs with a size below 15 nm demonstrated superparamagnetism. It also showed hysteresis loops for ferromagnetism, as shown in Figure 3a. The behaviors of the low Ms of the SiO_2_@Fe/SiO_2_ composite (FeCl_3_·6H_2_O at 0.1 M) may result from the low amount of Fe (0.59%) deposited on the surface of the spheres and a thin layer of the SiO_2_ shell coating. A greater amount of Fe (2.42%) deposited on the surface of the spheres made by FeCl_3_·6H_2_O at 0.375 M possessed higher Ms of the SiO_2_@Fe/SiO_2_ composite, shown with the red line in Figure 3a. It appears that 0.375 M of FeCl_3_·6H_2_O was the best to synthesize metallic particles. The SiO_2_@Fe/SiO_2_-0.375 composite spheres could be well dispersed and stored in an absolute ethanol solution, as shown in Figure 3b (left). All of the particles were attracted to the side of the magnet when the permanent magnet was placed outside of the vial, resulting in a clear solution (Figure 3b, right)). This observation confirms that the particles are in a magnetic state. Since the SiO_2_@Fe/SiO_2_-0.375 composite spheres exhibited the highest Ms value, these spheres were used for the subsequent dye release experiments and cell culture studies. SiO_2_@Fe/SiO_2_-0.375 composite spheres have been simply referred to as SiO_2_@Fe/SiO_2_ in the following sections.

### 3.2. Drug Release Profile of SiO_2_@Fe/SiO_2_ Nanoparticles

**Figure 4:**
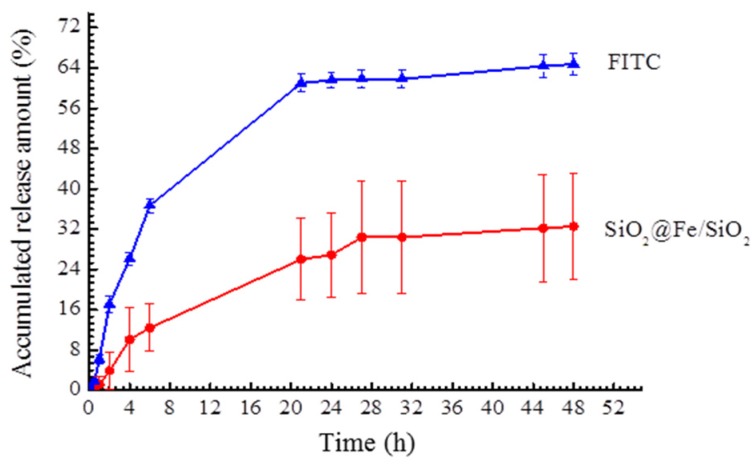
FITC loaded in SiO_2_@Fe/SiO_2_ composite spheres to examine the dye (FITC) release behavior for drug release applications (soaking in PBS at pH 7.4, 37 °C).

Figure 4 shows the FITC release profile of the SiO_2_@Fe/SiO_2_ composite spheres. Only FITC solution showed rapid release in the first 20 min (≈62%) using the dialysis method; but in SiO_2_@Fe/SiO_2_, the FITC originally loaded in the composite spheres was gradually released from the first 5 to 45 min, and its release rate increased from 10% to 28%. The FITC may be trapped in the mesoporous silica; it is not easy to diffuse from the dye releasement. The slow release pattern of SiO_2_@Fe/SiO_2_ is revealed in this test, and it shows the possibility of using SiO_2_@Fe/SiO_2_ composite spheres as a chemo drug carrier for long-term release.

### 3.3. In Vitro Evaluation of SiO_2_@Fe/SiO_2_ Composite Spheres

#### 3.3.1. Cell Viability and Cytotoxicity

**Figure 5:**
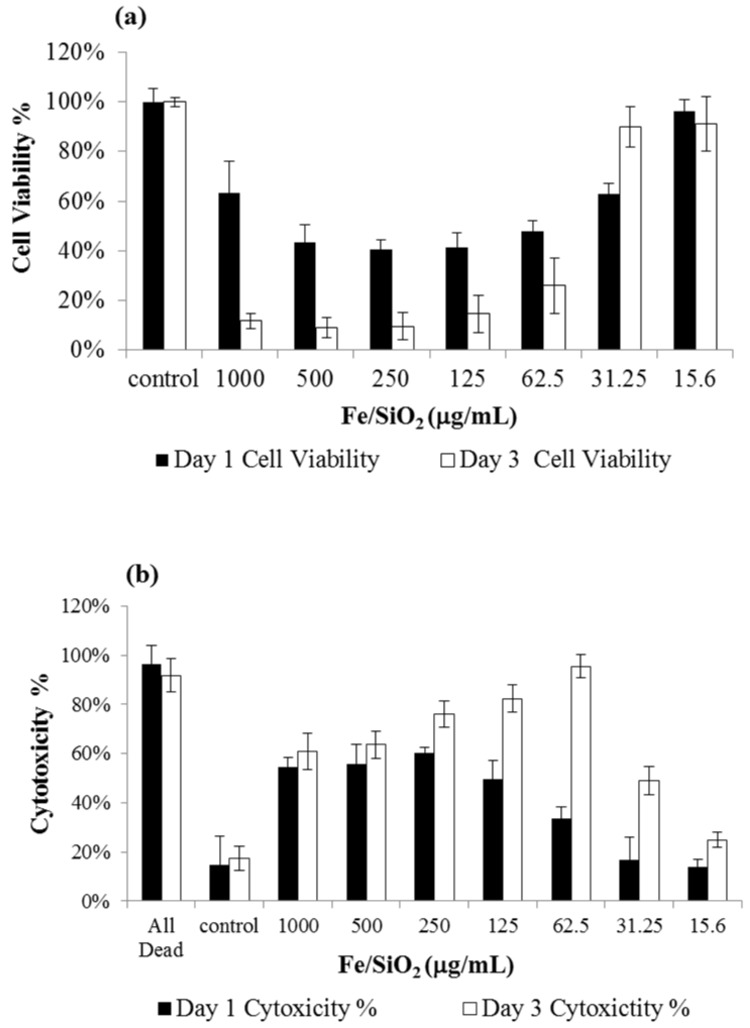
Results of (**a**) CCK-8 and (**b**) LDH assays of L929 cells after incubation with SiO_2_@Fe/SiO_2_ particles at different concentrations. Data were analyzed by the Student’s *t*-test and are presented as the mean ± SD; *n* = 6, * *p* < 0.05).

The cell viability and cytotoxicity results of L929 fibroblasts cells are shown in Figure 5. In Figure 5a, as the concentration of the SiO_2_@Fe/SiO_2_ composite spheres decreases, the cell viability increases. When the concentration is 15.6 μg/mL, the cell viability is about 100%, which is almost the same as that of the control groups. The results of the cytotoxicity assay are shown in Figure 5b. Significant differences are observed between the control group and the experimental group at higher concentrations (>31.25 μg/mL) of the SiO_2_@Fe/SiO_2_ composite spheres after Day 1 and Day 3 of co-culture. Only the lowest treatment concentration tested (15.6 μg/mL) shows non-toxicity to L929 cells. The LDH result is consistent with CCK-8 data that demonstrate that the fabricated SiO_2_@Fe/SiO_2_ composite spheres exhibit a non-toxic effect in L929 cells at a concentration of 15.6 μg/mL.

**Figure 6:**
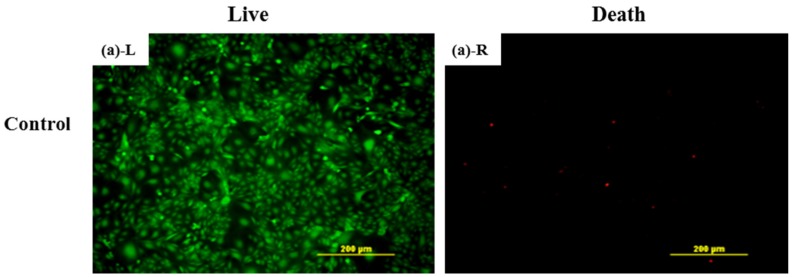

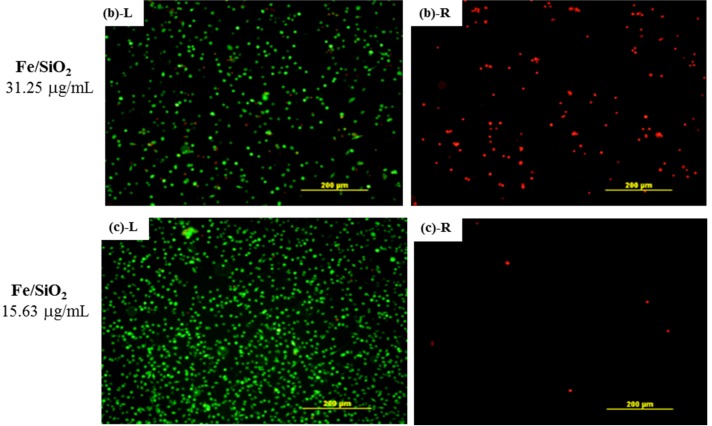
Live/dead stain results of L929 cells cultured with (**a**) medium only as the control and with SiO_2_@Fe/SiO_2_ spheres at (**b**) 31.25 μg/mL and (**c**) 15.63 μg/mL. (Left column showing the green fluorescence represents live cells; right column with red fluorescence reveals dead cells).

Figure 6 shows representative images of cells labeled with the live/dead stain. The live cells emit green fluorescence (left column), and the dead cells emit red fluorescence (right column) . In the control group (Figure 6a), a bright green fluorescence can be observed in the L929 cells, with only a few red-stained nuclei. These results indicate that the cells were mostly viable in the absence of the test materials. When cultured with 31.5 μg/mL of SiO_2_@Fe/SiO_2_ composite spheres, we observe a vast majority of live L929 cells, with some red-stained nuclei, indicating cell death, also being observed (Figure 6b); however, the morphology of cells was abnormal. At lower particle concentrations (16.25 μg/mL), an increased number of live cells with fewer dead cells (few red spots) was seen, indicating that SiO_2_@Fe/SiO_2_ composite spheres were nontoxic to L929 cells at low concentrations (Figure 6c).

#### 3.3.2. Intracellular Distribution of Particles

**Figure 7:**
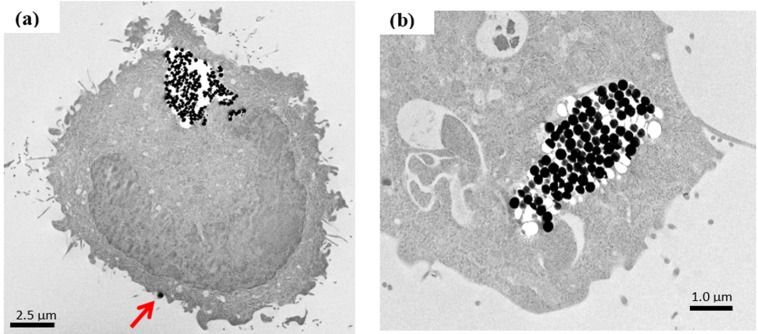
TEM micrograph of SiO_2_@Fe/SiO_2_ spheres’ uptake by L929 cells. Internalization by endocytosis (**a**) and distribution in cytoplasm (**b**) were observed.

To understand the uptake and distribution of SiO_2_@Fe/SiO_2_ composite spheres in cells, the L929 cells were co-cultured with the composite spheres for 2 h and then examined using TEM. The SiO_2_@Fe/SiO_2_ composite spheres were found to adhere to the cell membrane (Figure 7a, red arrow). The membrane cavity with many SiO_2_@Fe/SiO_2_ nanoparticles is shown (Figure 7a). No such electron-dense features, consistent in appearance with the NPs, were seen in the TEM images of the control cultures. Endosomes containing numerous SiO_2_@Fe/SiO_2_ composite nanospheres were revealed, too (Figure 7b), confirming the uptake of the composite spheres via an endocytosis process.

## 4. Conclusions

Fe/SiO_2_ composite spheres covered with a thin SiO_2_ protection layer (SiO_2_@Fe/SiO_2_) were successfully prepared in this study. It was determined via XRD analysis that nanoparticles were made of metallic iron, and not an oxidized form. The Fe content in the SiO_2_@Fe/SiO_2_ composite spheres was measured by ICP-MS to be 2.42 wt% when prepared using 0.375 M FeCl_3_·6H_2_O and exhibited the strongest magnetization (Ms 9.37 emu/g). From the dye (FITC) release profile, it was confirmed that SiO_2_@Fe/SiO_2_ composite spheres can act as a drug carrier. The results of the *in vitro* tests revealed that the SiO_2_@Fe/SiO_2_ composite spheres inhibited cell proliferation at a higher concentration (>16.25 μg/mL), but were nontoxic when at low concentrations of 16.25 μg/mL. The TEM results revealed that SiO_2_@Fe/SiO_2_ composite spheres were uptaken into the cytoplasm and retained in the endosome. A large concentration of these spheres caused cell death. The above results demonstrate that SiO_2_@Fe/SiO_2_ composite spheres, which are in a magnetic state and exhibit a slow release behavior, can act as a dual-functional agent in cancer therapy applications as hyperthermia and chemo agent carriers.
